# Single-Cell Transcriptomics Reveals Cellular Heterogeneity and Drivers in Serrated Pathway-Driven Colorectal Cancer Progression

**DOI:** 10.3390/ijms252010944

**Published:** 2024-10-11

**Authors:** Jiahui Wang, Yu Zhang, Xinyi Chen, Qi Sheng, Junluo Yang, Yuyao Zhu, Yuhang Wang, Fangrong Yan, Jingya Fang

**Affiliations:** School of Science, China Pharmaceutical University, Nanjing 211198, China; 15852911218@163.com (J.W.); zhangyu009351@163.com (Y.Z.); xychen0109@163.com (X.C.); 3322051464@stu.cpu.edu.cn (Q.S.); yjl1520207503@126.com (J.Y.); 3222051453@stu.cpu.edu.cn (Y.Z.); yuhang.w.7773@foxmail.com (Y.W.)

**Keywords:** colorectal cancer, scRNA-seq, serrated pathway, precancerous lesions, cancer progression

## Abstract

Serrated lesions are common precancerous pathways in colorectal cancer (CRC), but the process by which they progress to malignancy remains unclear. We aimed to elucidate this progression through a single-cell RNA landscape. We conducted single-cell RNA sequencing on three normal colonic tissues and fifteen SLs (including HPs, SSLs, SSLD, and TSAs) and integrated these data with datasets containing tumor samples. We identified three invasive malignant epithelial cell subtypes related to CRC progression: SLC1, SLC2, and tumor cell. SLC1, specific to SSLs, is involved in cell proliferation and shows a continuum of malignancy in gene expression. TSA-specific SLC2 exhibited FOXQ1 upregulation and active EMT, indicating invasiveness. The trajectory analysis showed that HPs do not progress to cancer, and different SL types are linked to the MSI status of advanced CRCs. We validated molecular drivers in premalignant lesions and later carcinogenesis. In the tumor microenvironment, CAF and pre-CAF fibroblast subtypes associated with progression were identified. During the premalignant stage, SLC1 triggered CD8+ T cell responses, while at the advanced stage, CAFs promoted tumor invasion and metastasis via FN1-CD44, influencing tumor progression and the treatment response. Our findings highlight transcriptional changes across serrated pathway stages, aiding in early CRC diagnosis and treatment.

## 1. Introduction

Colorectal cancer (CRC) ranks as the third most common malignant tumor worldwide [1]. The high mortality rate of CRC is closely related to its complex molecular mechanisms, strong heterogeneity, and invasiveness, making clinical treatment extremely challenging [2]. Conventional treatments like surgery, chemotherapy, and radiotherapy have limited success due to intratumoral heterogeneity [3]. Immunotherapy benefits only a small fraction of patients, necessitating new strategies targeting epithelial and stromal cells in the tumor microenvironment. A comprehensive understanding of the CRC ecosystem and the driving factors of cancer evolution is essential for discovering effective treatments. CRC is an ideal system to study the continuum of phenotypic states along malignant transformation because it follows a stereotyped progression from normal to precancerous polyps and then to cancerous ones [4]. The process of polyp carcinogenesis occurs through two main pathways: the traditional adenoma–carcinoma sequence and the serrated pathway, with serrated lesions (SLs) accounting for 15% to 35% of sporadic CRC precancerous lesions [5]. The serrated pathway, including hyperplastic polyps (HPs), sessile serrated lesions (SSLs), SSLs with dysplasia (SSLDs), and traditional serrated adenomas (TSAs), progresses faster than the traditional sequence [6]. However, the serrated morphology disappears in late stages, complicating the distinction between lesions from serrated or traditional pathways [7].

Transcriptomic studies using bulk RNA-seq and single-cell RNA sequencing (scRNA-seq) have explored CRC. Bulk RNA-seq measures the average gene expression in tissues, obscuring cell-specific profiles [8]. ScRNA-seq provides a single-cell resolution, identifying and characterizing subclusters with unique biological effects, which are crucial for understanding tumor progression mechanisms [9]. CRC originates from mucosal epithelial cells, and their heterogeneity and interactions within the tumor microenvironment are key to tumor development [10]. While these studies enhance our understanding of CRC’s transcriptional characteristics and microenvironment, gaps remain in exploring epithelial cell heterogeneity, identifying the subtypes driving cancer progression, and understanding their interactions with the tumor microenvironment.

In cancer progression research, identifying genes and pathways driving invasive cancer is crucial [11]. However, most studies focus on late-stage colon tumors, neglecting early events in precancerous lesions. Understanding the pathogenesis of serrated tumors in these lesions is essential for precision treatment and early intervention in CRC. Few studies address the invasive process of polyps transforming into malignant tumors, leaving gaps in understanding phenotypic changes and molecular drivers from normal to cancerous states [12].

We merged two batches of single-cell transcriptomic datasets, which included normal colon tissues, colon tissues with serrated lesions (SLs), and tumor colon tissues, to study the malignant transformation process of CRC. Through scRNA-seq, we aim to detail transcriptional profile changes from normal to precancerous and cancerous states, identifying malignant cell subtypes associated with CRC progression. Utilizing advanced techniques and algorithms, we explore developmental trajectories and molecular drivers of malignant subpopulations, revealing CRC’s invasive pathways. The goal is to enhance the evolutionary map of CRC progression and provide insights into the tumor microenvironment, supporting the early identification and precision treatment of CRC.

## 2. Results

### 2.1. ScRNA-Seq Atlas and Cellular Composition of Stepwise Progression of CRC

**Figure 1:**
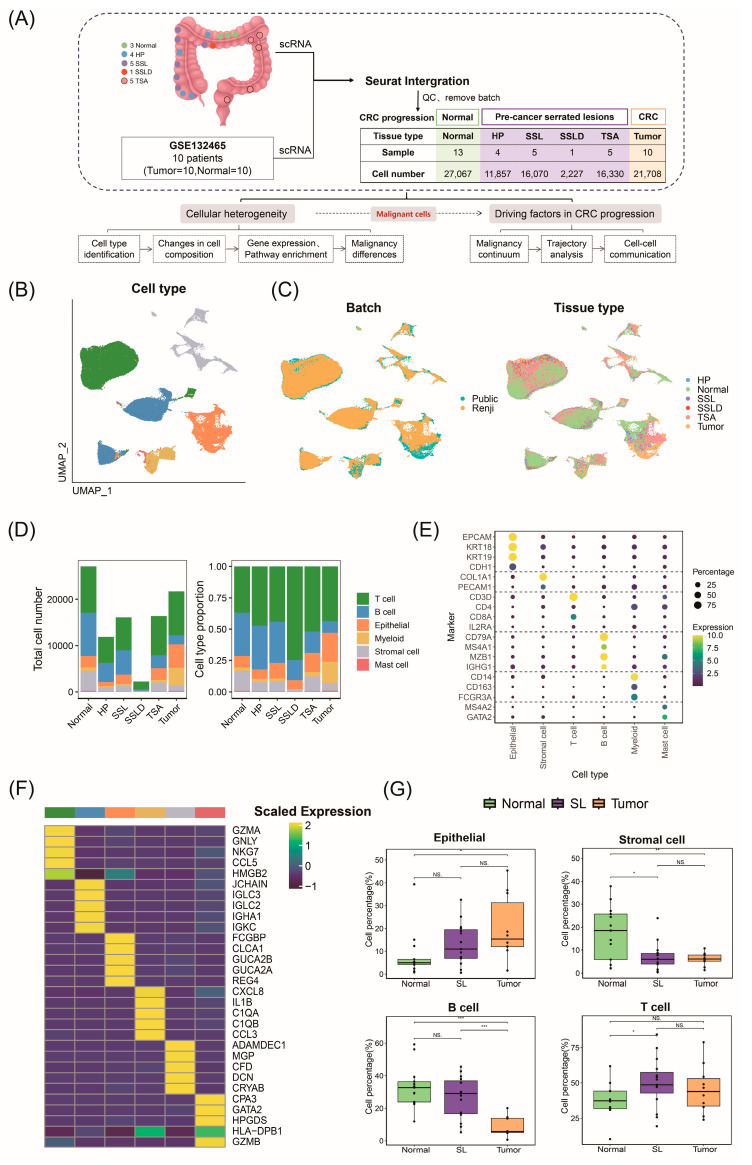
Single−cell transcriptomic atlas and cellular composition in progressive development of colorectal cancer (**A**) Flow chart of sample collection and processing. (**B**) Uniform manifold approximation and projection (UMAP) plot with cell type annotations. (**C**) Batch source plot (left) and tissue source plot (right) (Public: GSE132465 cohort; Renji: Renji Hospital data). (**D**) Bar chart showing distribution of cell numbers and proportions by tissue source. (**E**) Bubble chart displaying expression of marker genes used for cell type annotation. (**F**) Top five differentially expressed genes identified in each cell type from differential expression analysis. (**G**) Changes in cell numbers during CRC progression with pairwise differential testing (SL: combined HP, SSL, SSLD, and TSA). NS *p* > 0.05; * *p* < 0.05; ** *p* < 0.01; *** *p* < 0.001.

We constructed separate single-cell transcriptomic data analysis pipelines for 18 human colorectal samples obtained from Renji Hospital and 20 samples selected from the GSE132465 dataset. We merged the processed seurat objects and applied uniform quality control standards. Batch effects between the two datasets were effectively removed (Figure 1A). Ultimately, we generated scRNA-seq profiles of 13 normal tissues, 15 SSLs (including 4 HPs, 5 SSLs, 1 SSLD, and 5 TSAs), and 10 tumor tissues (Figure 1B,C and Figure S1). Based on differential expressions of known cell type marker genes, we identified six cell types (Figure 1E). The top 5 differentially expressed genes (DEGs) between cell types further confirmed the accuracy of the clustering and annotation results (Figure 1F).

To understand transcriptional changes in CRC progression, we compared cell abundances and compositions at different pathological stages (Figure 1D,G). As CRC progresses, the proportion of epithelial cells gradually increases, with proliferation rates exceeding those of normal cells, which is consistent with CRC pathogenesis originating from colonic mucosa epithelial cells. The regulation of epithelial cell proliferation and differentiation plays a crucial role in the onset and progression of CRC [13]. Recent studies have shown the enrichment of myeloid cells and specific types of T cells in CRC, while B cells are depleted [14]. We observed similar changes in the tumor immune composition, with B cells gradually decreasing. Previous studies have found a decrease in activated B cells in the primary lesions of CRC liver metastases, effectively inhibiting metastasis [15]. T cells are enriched in precancerous polyps but decrease in tumor tissues, indicating an adaptive immune response in the precancerous stage, followed by immune evasion in cancer [16]. T cell exhaustion, characterized by reduced cytokine production and increased inhibitory receptor expression, is a major mechanism of cancer immune evasion [17].

### 2.2. Invasive Malignant Epithelial Cell Subtypes Associated with CRC Progression

**Figure 2:**
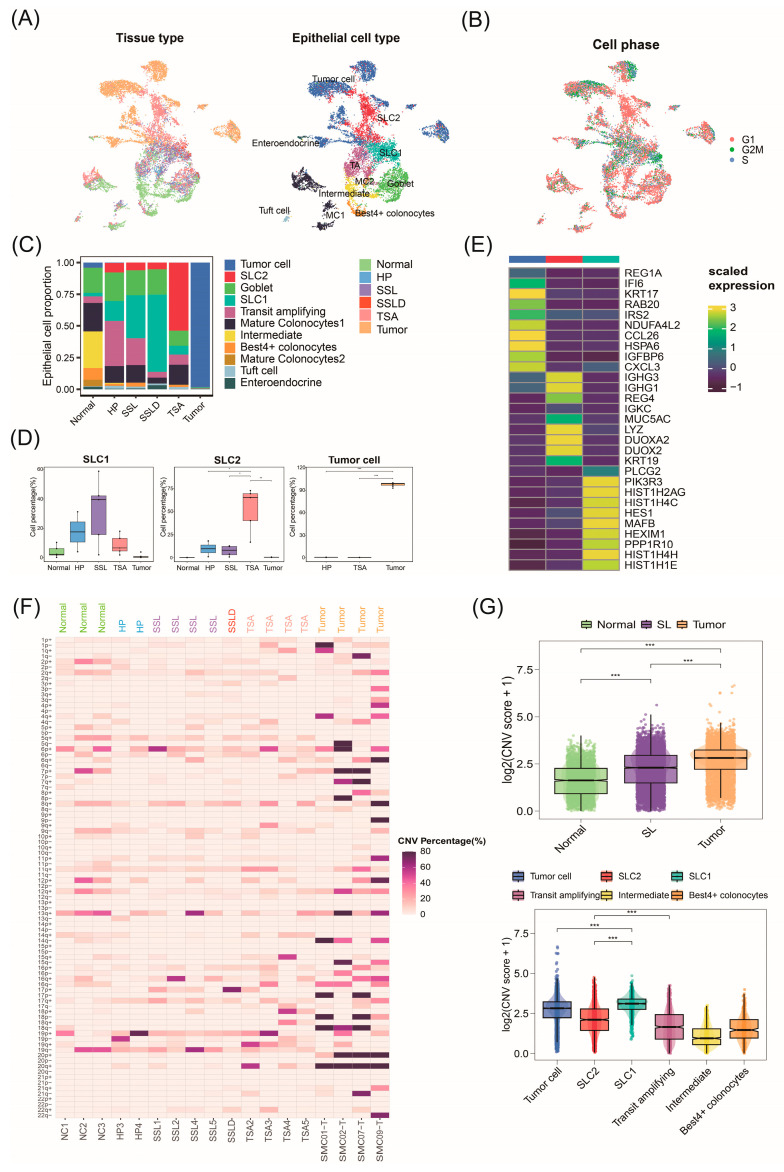
Identifying malignant cell subtypes associated with cancer origin in epithelial cells. (**A**) Tissue source of epithelial cells (left); UMAP plot with cell type annotations (right). (**B**) Cell cycle mapping in UMAP. (**C**) Bar chart showing distribution of cell numbers by tissue source. (**D**) Changes in cell numbers during CRC progression with pairwise differential testing. (**E**) Top 10 differentially expressed genes identified in each cell type. (**F**) Heatmap showing percentage of amplification and deletion in chromosomes 1–22 inferred for each individual from 18 samples. (**G**) Copy number variation (CNV) scores from interCNV for different tissue stages (top) and cell types (bottom). SL: serrated lesions (HP, SSL, SSLD, TSA). * *p* < 0.05; ** *p* < 0.01; *** *p* < 0.001.

CRC originates from mucosal epithelial cells of the colon, progressing invasively from the inside out. Analyzing epithelial cells is crucial for understanding CRC origin and progression. By re-clustering 13,139 epithelial cells and annotating based on marker gene expression, we identified eight normal epithelial cell subtypes. In addition, clusters 0, 5, 8, 9, 10, 12, 14, 16, and 18 mainly derived from tumor tissues and exhibited tumor cell marker genes, thus defined as tumor cells (Figure 2A and Figure S2B). Clusters 1, 3, and 19, primarily from serrated lesions (SLs) and some normal tissues, spanned stages from normal to polyps, indicating their role in precancerous progression.

To further determine the proliferation ability of these epithelial cells, we calculated the cell cycle scores for each cell and predicted their classification into G2M, S, or G1 phases. The results show cluster 3 cells in G2M and G1 phases, while clusters 1 and 19 were mainly in G1 (Figure 2B and Figure S2A). Given the high heterogeneity within the cell clusters, cluster 3 was defined as Serrated Lesion Cell 1 (SLC1), and clusters 1 and 19 were defined as Serrated Lesion Cell 2 (SLC2). SLC2, mainly from TSA tissues, is associated with TSA progression. SLC1, primarily from HP, SSL, and SSLD, constitutes most epithelial cells in SSLD, indicating its role in SSL progression (Figure 2D and Figure S2C). These SLC1 and SLC2 classifications are similar to previous subtype results [12].

Using the “FindAllMarker” function, we identified differentially expressed genes for each cell type compared to other cell types (Table S1). SLC1 highly expressed *PIK3R3*, which can inhibit cell senescence through the p53/p21 signaling pathway and promote cell proliferation [18]. Additionally, the SLC2 cell subtype highly expressed lysozyme (*LYZ*), a known Paneth cell marker primarily present in the small intestine and nearly absent in normal colonic epithelial cells, which is consistent with previous studies in the literature identifying *LYZ* as a diagnostic biomarker for TSA [12]. Tumor cells highly express the *CXCL3* gene. Studies have shown that targeting the CXCL3-CXCR2 signaling pathway to restore *IRF2* expression or therapeutically inhibit MDSCs can increase CRC sensitivity to immune checkpoint blockade (ICB) therapy [19]. Furthermore, a high expression of *REG1A* in tumor cells is also a marker of poor prognosis in CRC (Figure 2E) [20].

### 2.3. Malignant Origins of CRC Identified by Copy Number Variation Analysis

Previously, we identified malignant cells associated with CRC precancerous lesions in epithelial cells, specifically SLC1, SLC2, and tumor cells. Here, we infer the copy number variations (CNVs) of epithelial cells based on large-scale somatic CNVs to distinguish the malignancy levels of cells. Using normal epithelial cells from normal samples as a reference, we inferred the CNV status of cells from polyp and tumor tissues (Figure S2E). We created a heatmap of the gain and loss percentages of the long arm (q) and short arm (p) of chromosomes 1–22 for 18 samples (Figure 2F). The results indicate extensive chromosomal CNVs in epithelial cells at the tumor stage, with CNV events increasing from the SL stage and peaking at the tumor stage. This suggests a progressive increase in chromosomal gain/loss events throughout CRC progression, validating the malignancy process in epithelial cells. Chromosomal variations in 5q, 18q, and 17p are typical in CRC.

To identify malignant origins within epithelial cells, we compared the CNV scores across different epithelial cell subtypes using violin plots, which showed significant differences (kruskal.test) (Figure 2G). The SLC1 cluster exhibited the highest CNV scores, with SLC2 also showing higher scores than normal subtypes, indicating their critical roles in serrated polyp formation and development. Tumor cells had significantly higher malignancy levels than normal cells, corroborating their critical role in later carcinogenesis stages. By extracting the CNV scores of SLC1 and SLC2, we merged HP, SSL, SSLD, and TSA into SLs to observe CNV changes across three pathological stages. The results show that the CNV scores of SLC1 and SLC2 gradually increased during CRC progression, providing strong evidence that SLC1 and SLC2 serve as origins of CRC.

### 2.4. The Biological Functions of Malignant Cell Subtypes Involved in CRC Progression

**Figure 3:**
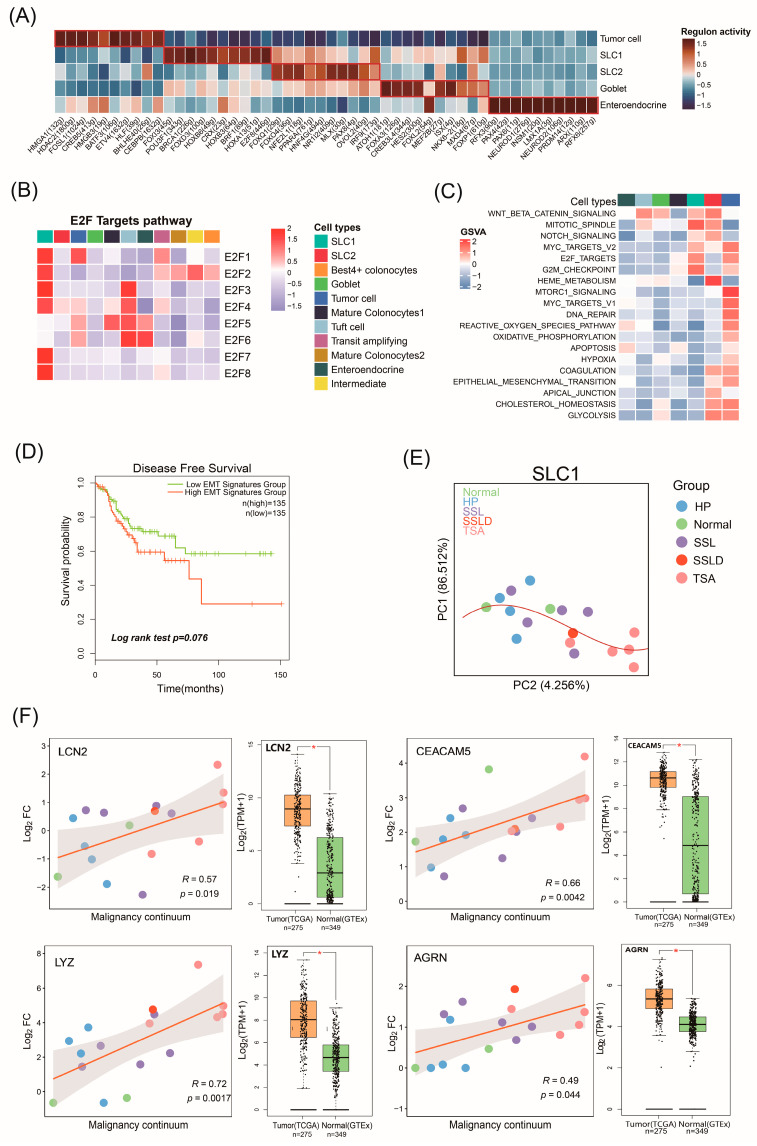
Functional and characteristic investigation of SLC1 in malignant continuum (**A**) Heatmap of transcription factor gene expression regulation in epithelial cells. (**B**) Heatmap showing expression of E2F family in each epithelial cell subtype. (**C**) GSVA showing enrichment scores of hallmark pathways among cell subtypes. (**D**) Survival analysis of high and low EMT groups in TCGA−COAD. (**E**) Malignant continuum of SLC1. Each dot represents one sample. PC1 and PC2 are based on log2 FC values between SLC1 cells from each sample and normal SLC1 cells, identifying significantly different genes (MAST test, differentially expressed in at least two samples). Spline is fitted to first two principal components (red), with samples ordered by their position along spline. (**F**) Scatter plot: correlation analysis between log2FC values and PC2 using Spearman method. Box plot: comparison of mRNA expression levels between tumor samples from TCGA−COAD and matched normal samples from Genotype−Tissue Expression (GTEx).The color of the scatter plot indicates the group from which each sample originates. * *p* < 0.05.

Single-Cell Regulatory Network Inference and Clustering (SCENIC) was employed to evaluate the differential expression levels of transcription factors (TFs) across epithelial cell subtypes. In SLC1, TFs such as *FOXI3*, *BRCA1*, *FOXD3*, *CRX*, *HOXB3*, *BRF1*, *HOXA13*, and *E2F8* were upregulated, while in SLC2, *FOXQ1*, *FOXO4*, *NFE2L1*, *PPARA*, and *HNF4G* showed higher expression, indicating distinct developmental pathways between SSL and TSA (Figure 3A). Notably, *E2F8*, a key regulator of cell division, was highly activated in SLC1, along with other E2F family members (*E2F1*-*E2F8*), highlighting SLC1’s role in cell proliferation (Figure 3B). The carcinogenic activation of the E2F transcription program can drive the proliferation of otherwise quiescent cells [21]. FOXQ1 was highly upregulated in SLC2. *FOXQ1* promotes tumor metastasis in CRC by inducing epithelial–mesenchymal transition (EMT) in cancer cells and can exacerbate cancer by activating the oncogenic Wnt/β-catenin signaling pathway [22].

A gene set variation analysis (GSVA) analysis corroborated these findings, showing the upregulation of the E2F_Targets pathway in SLC1 and the EMT and Wnt/β-catenin pathways in SLC2 (Figure 3C). The NOTCH signaling pathway was significantly upregulated in both SLC1 and SLC2. Additionally, tumor cells exhibited the activation of oncogenic and metabolic features like Myc and the G2M checkpoint. The EMT pathway was progressively upregulated during the transition from SLC2 to tumor cells, indicating that TSA lesions undergo an active EMT process. EMT is considered a crucial step for tumor cell invasion, and metastasis and is associated with tumor stem cell properties, including self-renewal and multipotency, thus maintaining a highly invasive and aggressive state [23]. A survival analysis of the TCGA-COAD cohort revealed a significant correlation between high EMT levels and poor prognosis in CRC (Figure 3D). These findings suggest that SLC1 and SLC2 cells are malignant cells driving CRC progression, albeit through different mechanisms. SLC1 is involved in active cell proliferation, while SLC2 promotes tumor cell invasion through the EMT process. For detailed data on the GSVA, refer to Table S3.

### 2.5. Potential Malignant Continuum Formed by SLC1

SLC1 and SLC2 cells are linked to the precancerous origins of CRC. SLC1 cells span normal and serrated polyp tissues, allowing us to explore the malignant progression in SSL polyps. By comparing gene expression in SLC1 cells from polyps and normal tissues, we identified abnormal gene expression changes. After calculating significant differences, we computed the principal components of these log2FC differences and sorted the samples based on their positions in the first two principal component spaces. The location of the samples can be interpreted as the position in the continuum from normal tissue to cancer (Figure 3E). This analysis indicates a gradual gene expression progression from normal tissue to early and then late polyps. We further explored specific gene expression changes along this continuum. The scatter plot represents the gene expression changes in each sample along the malignant continuum. We validated this by combining bulk mRNA data from 275 tumor samples from TCGA-COAD and 349 normal samples from the GTEx database to explore whether the gene expression differences between normal and tumor samples align with the continuum analysis results (Figure 3F). A Logrank survival analysis in TCGA-COAD identified 62 genes significantly impacting survival, listed with correlation and survival results (Table S2).

Notable genes include *LYZ*, *LCN2*, *CEACAM5*, and *AGRN*. *LYZ* expression gradually increased along the malignant continuum, representing cell differentiation trends. *LCN2*, a confirmed oncogene in CRC, and *CEACAM5*, a common tumor marker in CRC diagnosis and treatment, both showed significantly increased expression along the continuum (*p* < 0.05) [24,25]. Box plots showed that the expression of *LYZ*, *LCN2*, and *CEACAM5* from external datasets was significantly higher in tumor samples compared to normal samples (*p* < 0.01), complementing our findings. AGRN-specific expression, confirmed by immunostaining, serves as a novel biomarker distinguishing SSL from HP and TSA [26]. Our results also show increasing *AGRN* expression in SLC1 cells during CRC progression, which is consistent with the conclusion that SLC1 cells are SSL-specific. Therefore, the *LYZ*, *LCN2*, *CEACAM5*, and *AGRN* genes, with specific expression along the malignant continuum, may serve as markers for CRC precancerous diagnosis and tumor progression detection.

### 2.6. Factors Driving CRC Progression Identified by Trajectory Analysis

**Figure 4:**
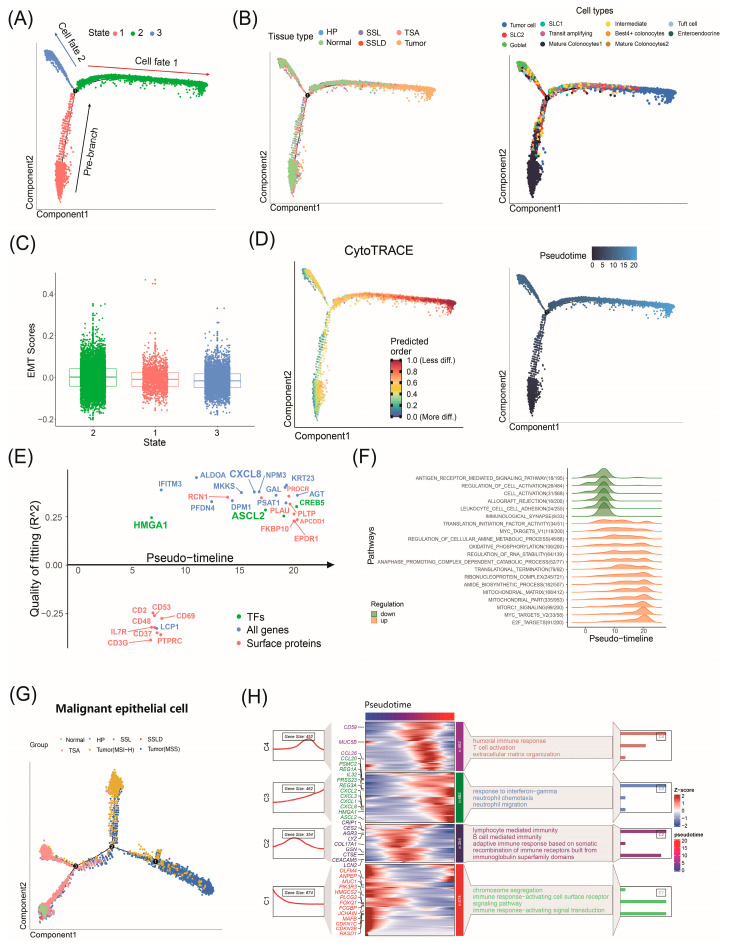
Trajectory analysis to investigate factors driving CRC progression. (**A**) Monocle trajectory analysis identified 3 states. Pre−branch represents root, with arrows indicating two cell fates (manually annotated). (**B**) Mapping of tissue origin, cell types in trajectory. The number 1 represents the first bifurcation fulcrum in the trajectory. (**C**) Box plot showing epithelia−lmesenchymal transition (EMT) scores of 3 states. (**D**) Mapping of CytoTRACE scores and pseudotime in trajectory. (**E**) GeneSwitches output showing ordering of switch genes along pseudotime. (**F**) Enrichment analysis of switch genes, covering hallmark, KEGG, and GO gene sets from MSigDB. (**G**) Group distribution along Monocle trajectory of malignant epithelial cells SLC1, SLC2, and tumor cells (distinguish tumors as MSI-H or MSS). (**H**) Heatmap of differentially expressed genes in malignant cells along pseudotime, including 1415 genes significantly influencing pseudotime (*p* < 0.05). Left curve shows gene counts, while right side highlights enriched pathways.

Using Monocle, we constructed a trajectory for all epithelial cells (Figure 4A). The tissue distribution on the trajectory showed that state 2 transitioned from normal tissue and SLs transitioned to tumors. State 1 and state 3 mainly consisted of cells from normal tissue and SLs (Figure 4B). SLC1 and SLC2 were primarily located in the middle of the trajectory, with tumor cells at the end of state 2, indicating potential differentiation into tumor cells. The density changes along the pseudotime trajectory demonstrated a consistent cell evolution process (Figure S3C).

We used EMT scores to infer the root and evolutionary path of the trajectory. Higher EMT scores in state 2 suggest that state 2 is in the later stages of CRC progression (Figure 4C). Based on the distribution of cell subtypes on the trajectory, Cell fate 1 represents a progression from normal epithelial cells to malignant tumor cells, termed the tumor branch. Cell fate 2 represents a transition from normal cells to SL-related cells without further progression, termed the SL branch. Notably, the results show that cells from HPs are primarily distributed in the SL branch, indicating that these cells do not participate in further carcinogenesis. This further supports the clinical fact that HPs are generally benign lesions and do not progress to cancer. A CytoTRACE assessment showed higher differentiation levels along Cell fate 1, indicating increasing malignancy (Figure 4D). High stemness cells, identified by CytoTRACE scores above 0.75, clustered at the end of the tumor branch (Figure S3A,B).

For the tumor branch (Cell fate 1), we used GeneSwitches to calculate the expression of key genes and the time of pathway activation and deactivation along the pseudotime analysis. We found that HMGA1 was activated early in the progression process, while ASCL2 and *CXCL8* were activated in the mid to late stages of the progression process (Figure 4E). HMGA1 is significantly associated with poor prognosis in CRC and promotes CRC invasion by increasing *GLUT3* transcription and expression [23]. ASCL2 is the master regulator of intestinal stem cell fate, and *CXCL8* is an inflammatory chemokine elevated in the CRC tumor microenvironment, playing a crucial role in promoting angiogenesis, invasion, and metastasis [27,28]. MYC_targets, E2F_targets, and oxidative phosphorylation pathways were also upregulated along pseudotime (Figure 4F). Using the Branch Expression Analysis Model (BEAM), we identified branch-dependent genes across three states. We identified 80 genes regulating cell differentiation from state 1 (Pre-branch) to state 2 (Cell fate 1) and state 3 (Cell fate 2) (Figure S3E). A trajectory analysis of SLC1, SLC2, and tumor subtypes highlighted significant driver genes at various stages of malignancy (Figure S3F). Within tumor cells, we identified the CMS1, CMS2, and CMS3 subtypes, indicating heterogeneity in progression. Separating tumor samples by MSI-H and MSS shows distinct branching at the trajectory’s end (Figure 4G). This suggests that SLC1 and SLC2 may lead to different microsatellite statuses in CRC. The findings support that TSA typically progresses to MSS CRC, while MSI-H CRC usually arises from SSLs. We found 1415 differential genes (*p* < 0.05) in the malignant cell pseudotime trajectory (Table S4) and performed an enrichment analysis (Figure 4H). Genes such as *LYZ*, *LCN2*, *CEACAM5*, *PIK3R3*, and *FOXQ1* drive CRC progression in precancerous lesions, while *CXCL8*, ASCL2, and HMGA1 drive early carcinogenesis, validating our conclusions from GeneSwitches, the malignant continuum analysis, and the functional analyses.

### 2.7. Transcriptomic Landscape Alterations in Stromal and Immune Cells throughout Progression of CRC

**Figure 5:**
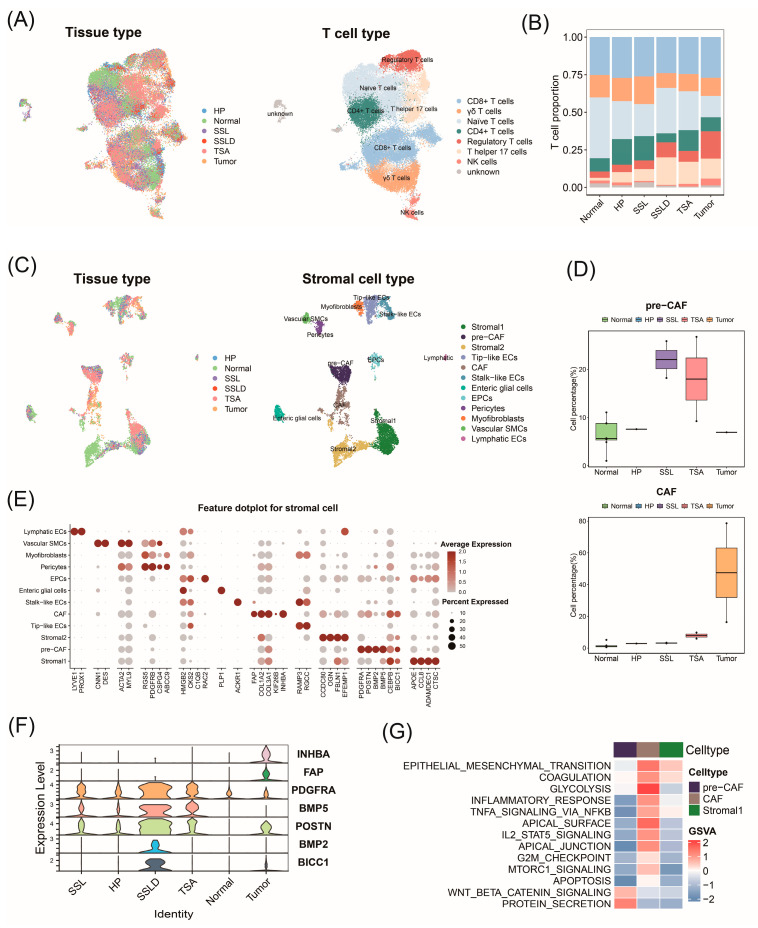
Transcriptional changes in stromal and immune cells during CRC progression. (**A**) Distribution of tissue origin in T cell UMAP plot (left); UMAP plot with T cell subtype annotations (right). (**B**) Bar chart showing distribution of T cell subtype proportions by tissue origin. (**C**) Distribution of tissue origin in stromal cell UMAP plot (left); UMAP plot with stromal cell subtype annotations (right). (**D**) Changes in numbers of pre−CAF and CAF during CRC progression with pairwise differential testing. (**E**) Bubble chart displaying expression of marker genes used for annotating stromal cell subtypes. (**F**) Expression of pre−CAF and CAF marker genes across different tissues. (**G**) GSVA showing enrichment scores of hallmark pathways among stromal cell subtypes.

In addition to focusing on the malignant epithelial cells driving CRC progression, we further investigated the role of the tumor microenvironment (TME) in CRC. We examined the single-cell transcriptomes of stromal and immune cells across CRC progression. By clustering T cell components and visualizing them with UMAP, we identified seven major T cell subtypes based on known markers (Figure 5A and Figure S4A,B and Table S5). Compared to normal colon samples, we observed a decrease in naive T cells and an increase in CD4+ T cells in tumors (Figure 5B).

We clustered 9690 stromal cells into 17 clusters, identifying enteric glial cells, pericytes, vascular smooth muscle cells, and various types of endothelial and fibroblast cells based on known marker genes (Figure 5C and Figure S4C, and Table S6). Among the fibroblasts, we observed a cluster of cancer-associated fibroblasts (CAFs) almost entirely composed of cells from tumor tissues. Another cluster of fibroblasts predominantly originated from normal colon tissue and the SL. Given the high expression of its marker genes, we referred to this cluster as pre-cancer-associated fibroblasts (pre-CAFs) (Figure 5D and Figure S4D). CAFs promote cancer initiation and progression through various mechanisms, including matrix remodeling, cell–cell interactions, and immune surveillance disruption [29,30]. The CAFs highly expressed the genes *FAP*, *COL1A2*, *COL3A1*, *KIF26B*, and *INHBA*, which are known as typical CAF characteristic genes. Among these, *FAP* and *COL1A2* were involved in extracellular matrix remodeling and were upregulated in various cancers (Figure 5E) [31,32,33]. This indicates that CAFs contribute to the unique ECM remodeling process within tumor tissues and drive CRC progression, which is absent in normal colon tissue or precancerous polyps. Pre-CAFs exhibited a high expression of genes like *PDGFRA*, *POSTN*, *BMP2*, *BMP5*, *CEBPB*, and *BICC1*, associated with the serrated pathway of CRC and cancer progression [34]. PDGFRA+ fibroblasts secrete MMP11, promoting growth factor (HBEGF) cleavage and SL development [35]. This suggests that pre-CAFs drive CRC progression by regulating cell proliferation and migration during the cancerous transformation. When comparing the distribution of these marker genes across tissues, it was seen that pre-CAF markers were highly expressed in SLs, whereas CAF markers were predominantly expressed in tumor tissues (Figure 5F). The expression of *PDGFRA*, *POSTN*, and *BMP5* is significantly increased in SSLD, making them potential biomarkers for the progression from SSL to SSLD. These findings indicate that phenotypically distinct fibroblasts exist in polyps and tumors, playing different roles in tumorigenesis. The GSVA revealed the enrichment of typical cancer-related pathways such as EMT, glycolysis in CAFs, and the Wnt/β-catenin pathway in pre-CAFs (Figure 5G). This suggests potential interactions between CAFs, pre-CAFs, SLC2, and tumor cells.

### 2.8. Cell–Cell Interactions during CRC Progression

**Figure 6:**
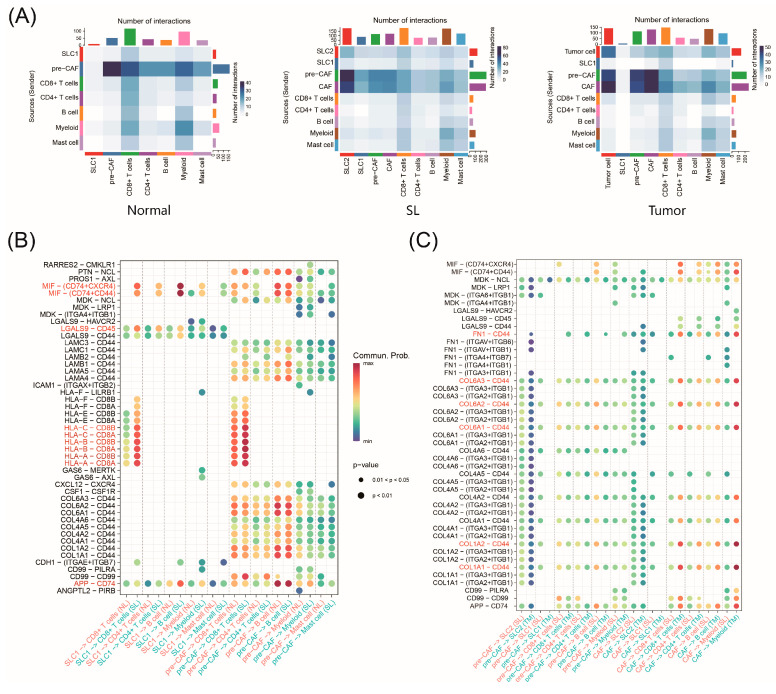
Cell–cell interactions during CRC progression. (**A**) Heatmap showing number of cell–cell interactions in normal, SL, and tumor samples. (**B**) Ligand-receptor pairs of cell–cell interactions in normal and SL samples. (**C**) Ligand-receptor pairs of cell–cell interactions in SL and tumor samples.

Growing evidence indicates that interactions between tumor cells and stromal cells in the TME play crucial roles in regulating tumor progression and treatment response [36,37]. To decipher the crosstalk between malignant cells (SLC1, SLC2, CAFs, and pre-CAFs) and other TME components during CRC progression, we used CellPhoneDB to identify potential signaling molecules based on ligand–receptor interactions. We evaluated cell interactions in normal, SL, and tumor groups (Figure 6A). The results show higher interaction numbers in SLs compared to normal tissues, suggesting that serrated polyps drive early tumorigenesis. Notably, CAFs emerged in the SL communication network, with increased interactions between pre-CAFs and other cells. In the tumor stage, the network included tumor cells, and CAFs had significantly increased interactions with other cells (Figure S4E).

A detailed receptor–ligand analysis revealed that, compared to normal tissues, SLC1 from SLs signaled more to B cells through MIF-CD74/CD44, MIF-CD74/CXCR4, and APP-CD74 (Figure 6B). Additionally, there were significantly more HLA-I ligand–CD8A/CD8B receptor pairs in SL tissues, indicating strong functional interactions between SLC1 and CD8+ T cells at this stage. The primary function of *HLA* is to present endogenous antigens to CD8+ T cells. In combination with *CD8A* and *CD8B* receptors, *HLA* can trigger a cell-mediated immune response, helping the immune system to effectively recognize, attack, and eliminate abnormal cells and pathogens [38]. Therefore, such immune response mechanisms might exist in the precancerous stage. Compared to CAFs from SLs, CAFs from tumors signaled more to CD8+ T cells and myeloid cells through FN1-CD44 (Figure 6C). As an extracellular matrix protein, FN1 binding to *CD44* may influence the invasive and metastatic capabilities of tumor cells. Additionally, CAFs strongly expressed *COL6A* and *COL1A* family genes, which interact with *CD44* and were widely present in the tumor stage.

## 3. Discussion

Identifying individuals in the precancerous stage for effective intervention is crucial for preventing cancer mortality. While bulk sequencing has extensively studied the colorectal adenoma–carcinoma sequence, single-cell transcriptome studies on epithelial cell evolution and molecular characteristics are scarce [39]. Most research has focused on late-stage tumors, with little research available on precancerous lesions, especially the serrated pathway. Our study addresses this gap by providing a high-resolution scRNA-seq atlas spanning normal, precancerous, and cancerous stages, capturing key transcriptional events in CRC development. These findings offer valuable insights into early CRC progression and potential targets for prevention, diagnosis, and treatment.

An analysis of the cellular composition during CRC progression revealed an increasing proportion of epithelial cells. In the precancerous stage, malignant cells SLC1 and SLC2 were predominant. As polyps developed into tumors, the epithelial compartment was dominated by tumor cells exhibiting distinct gene expression programs and enrichments compared to other cells. Changes in stromal and immune cell composition and states, influencing the tumor microenvironment, were also identified. Within the stromal compartment, pre-CAFs predominated during the serrated precancerous stage, while CAFs became dominant as polyps progressed into tumors.

Tumor cells exhibit significant heterogeneity [40]. Different epithelial cell subtypes in our samples exhibited unique molecular characteristics. We identified two new malignant cell subtypes, SLC1 and SLC2, representing SLs with distinct biological characteristics. Among them, SLC1 are SSL-specific cells, and SLC2 are TSA-specific cells. SLC1 was involved in cell proliferation, forming a malignant continuum in gene expression. Genes like *LYZ*, *LCN2*, and *CEACAM5* showed significant expression increases along this malignant continuum and could serve as markers for SSL progression. A significant feature of TSAs is the presence of R-spondin fusions or RNF43 mutations, which are closely associated with the activation of the Wnt signaling pathway [41]. Our data further support and validate this conclusion. SLC2 upregulates *FOXQ1*, promoting metastasis through EMT and activating Wnt/β-catenin signaling pathways.

We mapped the differentiation trajectories of malignant cells in CRC progression. HPs are the most common and are usually considered benign, with MVHPs potentially progressing to SSLs [42]. Our results also show that while some HPs can differentiate into SSLs, they do not progress to CRC. SSL-specific SLC1 and TSA-specific SLC2 have the potential to differentiate into tumor cells. Specifically, different serrated lesions may lead to CRCs with different microsatellite statuses: TSA typically progresses to MSS CRC, while MSI-H CRC usually arises from SSL. Gene HMGA1 plays a driving role in the early stages of CRC progression. The ASCL2 and *CXCL8* genes might be key regulators of malignant transformation. A pseudotime analysis revealed 1415 differentially expressed genes, which are useful for exploring survival-related genes in bulk RNA datasets or constructing prognostic models.

In the TME analysis, we mapped stromal and immune cell scRNA profiles during CRC progression, investigating cell–cell communication. Stromal cells play a significant role in the formation of serrated tumors [35]. We identified pre-CAF and CAF, which are related to CRC progression. Pre-CAFs and CAFs exhibit different phenotypes and play distinct roles in tumorigenesis. Pre-CAFs regulate cell proliferation and migration during carcinogenesis, while CAFs remodel the extracellular matrix, driving the polyp-to-tumor transition. *PDGFRA*, *POSTN*, and *BMP5* are biomarkers for the progression from SSL to SSLD. Pre-CAF and CAF actively interact with other components in the tumor microenvironment. In the precancerous stage, SLC1 triggers CD8+ T cell immune responses. During tumor progression, CAFs facilitate infiltration and metastasis via FN1-CD44 interactions.

## 4. Materials and Methods

### Dataset Preparation and Processing

In this study, all of the data were downloaded from public databases. We obtained scRNA-seq data of eighteen human colorectal samples from Renji Hospital, School of Medicine, Shanghai Jiao Tong University, including four HPs, five SSLs, one SSLD, five TSAs, and three normal samples. We previously conducted related studies using this dataset, and the data were published in the Genome Sequence Archive (GSA) under accession number HRA002611. Additionally, we obtained scRNA data from Gene Expression Omnibus (GSE132465), including 10 normal and 10 matched primary tumor samples from patients with CRC. The tumor samples covered stages I-III and the four CMS subtypes. The detailed information of the other methods used in the study, including the copy number variation estimation, gene set variation analysis, SCENIC analysis, definition of malignancy continuum, trajectory analysis, and cell–cell interaction network analysis can be found in Doc S1.

## 5. Conclusions

In summary, our study focused on several key aspects of CRC progression: (1) dynamic transcriptional and cellular composition changes from the serrated pathway to CRC; (2) the identification of new malignant cell subtypes initiating different SLs; (3) epithelial cell heterogeneity in SSLs and TSAs; (4) CRC progression trajectories and driving factors; and (5) the construction of the stromal and immune cell atlas within the TME and the elucidation of the cell–cell interactions driving CRC progression. Although some SLs are managed with endoscopy and usually require no further treatment, our findings are valuable for precision prevention and personalized treatment. We clarified the evolutionary roadmap of CRC driven by different serrated lesions and identified key biomarkers and pathways at various stages, aiding in early detection and disease progression assessment. Developing personalized treatment strategies by targeting these pathways could be a future focus.

One limitation of our study is sample representativeness. Firstly, clinical constraints prevent the longitudinal collection of primary tumor tissues at multiple progression points. Additionally, due to limited SL tissue availability (<1 cm), mutation testing was not conducted despite known associations (e.g., *BRAF* mutations in 60–80% of SSLs and *KRAS* in 50% of TSAs) [43]. In the future, larger studies integrating genomics, transcriptomics, and proteomics are needed to comprehensively study CRC pathogenesis, progression, and treatment.

## Data Availability

The data used to support the findings of this study are available from the corresponding author upon request.

## Supplementary Materials

The following supporting information can be downloaded at https://www.mdpi.com/article/10.3390/ijms252010944/s1.
