# Supplementary material for: Single-Cell Transcriptomics Reveals Cellular Heterogeneity and Drivers in Serrated Pathway-Driven Colorectal Cancer Progression
Source: Int J Mol Sci. 2024 Oct 11;25(20):10944. doi: 10.3390/ijms252010944 (PMC11507054; doi:10.3390/ijms252010944)
Supplement: Supplementary file 1 [file ijms-25-10944-s001.zip › Doc.S1.pdf]

Doc. S1 Supplementary materials and methods

1 Single-cell RNA sequencing analysis and identification of marker genes

For the scRNA datasets from Renji Hospital and the public dataset GSE132465, they were each converted into Seurat objects using the Seurat R package (version 4.1.0), and then merged them using the “merge” function. High-quality cells were selected based on the same quality control criteria: 600-4,000 genes and fewer than 40,000 UMIs, with UMIs that were derived from the mitochondrial genome not exceeding 20%. We then used the “RunPCA“ function in the R package Seurat to perform principal component analysis on the dataset to reduce dimensionality. This method projects the normalized matrix onto 50 principal components, which capture nearly all the information in the dataset. Subsequently, the Harmony algorithm was applied to remove batch effects present in the principal components, using a method that maximizes batch sample diversity and iterative convergence. This step aimed to avoid overfitting and ensure that batch effects between tumor and normal tissue samples were minimized. For the Harmony analysis results, the top 30 components with the highest explanatory variance were selected for downstream analysis. Further, the “RunUMAP” function was applied to the Harmony-debatched results using UMAP dimensionality reduction to visualize the dataset in 2 dimensions. Clustering was performed using the “FindCluster” function, and the “FindAllMarkers” function was utilized to compare cells within a specific cluster to all other clusters, requiring marker genes to have an average expression in a specific cluster that was 2.5 times higher than in other clusters. We combined semi-supervised automated annotation with manual annotation of typical biological markers for cell type classification. For the semi-supervised automated annotation, we used the R package "SingleR". This approach allows for semi-supervised identification of cell types at the single-cell level. Finally, we determined consensus cell type annotations by examining the expression distribution of typical biological markers reported in the literature across each cluster. Cell cycle stages were predicted using the CellCycleScoring function to calculate the cell cycle score for each cell and classify cells into G2M, S, or G1 phase.

2 Copy number variation estimation

InferCNV (version 1.10.1) was used to estimate copy number variations in epithelial cells based on the transcriptome profile, using CNV scores from epithelial cells in normal samples as reference. The following parameters were used: “denoise = T”, “HMM = T”, “cutoff=0.1”. To reduce the possibility of false positives, a default Bayesian latent mixture model was used for CNV calling to identify the posterior probabilities of changes in each cell. CNVs with low probabilities were filtered out using the default threshold of 0.5. To determine the clonality of CNV changes in each tumor, the “subcluster” method on CNVs generated by HMM was utilized. CNVs were converted to changes at the p- or q-arm level using the GRCh38 cytoband information to simplify their positions. Each CNV was annotated as gain or loss, and the CNV score for each cell was the quadratic sum of CNV regions.

3 Gene Set Variation Analysis of cell subtypes

The GSVA package (version 1.42.0) in R was utilized to investigate the most enriched hallmark pathways in the cell subtypes of interest obtained from the Molecular Signatures

Database (https://www.gsea-msigdb.org/gsea/msigdb/index.jsp). The ComplexHeatmap package in R (version 2.15.4) was employed to generate a heatmap of the relevant biological pathways.

4 SCENIC analysis

The pySCENIC (version 0.11.0) package was used to identify gene regulatory networks and key transcription factors (TFs) in scRNA-seq data. This process includes inference of TF co-expression modules, enrichment analysis, calculation of regulon activities, and determination of regulon specificity. In this study, key TFs in epithelial cell subtypes were predicted based on target gene weights and transcriptional profiles, providing insights into transcriptional regulation in the progression of CRC.

5 Definition of malignancy continuum and identification of differential genes along continuum

To compute the malignancy continuum, we computed differential genes between SLC1 cells from each sample and SLC1 cells from normal samples. Eleven normal samples containing a sufficient number of SLC1 cells was selected as reference samples. The "FindMarkers" function in Seurat was utilized, with ident.1 set as the sample of interest and ident.2 as the reference sample. Parameters were configured as follows: "min.pct = 0", "logfc.threshold = 0", "min.cells.feature = 0", and "max.cells.per.ident = 300". Model-based Analysis of Single-cell Transcriptomics (MAST) was used as the differential testing. Gene expression differences between SLC1 cells from each sample and the selected reference were obtained, along with the corresponding log2FC values. The significance cutoffs for including a gene were MAST Padj $\leqslant$ 0.05, ∣ log2 FC ∣ $\geqslant$ 0.5 and in $\geqslant$2 samples.The "prcomp" function in R was utilized to calculate the principal components of the log2FC values of significant differential genes. The top two principal components PC1 and PC2 were selected as the x and y axes for plotting a scatter plot of the samples. The spatial distribution of the samples was interpreted as their position along the malignancy continuum from normal tissue to tumor, and a nonlinear fit was applied. In the identification of differential genes along continuum, the correlation analysis between the log2FC values of differential genes and PC2 was performed using the Spearman method.

6 Analysis based on public databases

We utilized independent datasets from The Cancer Genome Atlas (TCGA) and Genotype-Tissue Expression (GTEx) as validation datasets. The TCGA-COAD cohort includes RNA-seq expression profiles and clinical information of 275 cancer patients, while the GTEx database's colon cohort comprises RNA-seq expression profiles of 349 normal samples. We obtained mRNA transcriptomic profiles in TPM values and clinical information of patients, converting TPM values to log2(TPM + 1), and applied the "ComBat" algorithm to remove batch effects.We selected genes obtained from the scRNA-seq data and performed differential expression analysis for these genes between the TCGA-COAD cohort and GTEx-colon cohort. For the genes and pathways of interest, we conducted survival analysis in TCGA-COAD. Based on the average expression of each patient, we divided them into high and low groups using the median. We performed survival analysis using the R packages

"survival" (version 3.2.3) and "survminer" (version 0.4.7), generating Kaplan-Meier survival curves for the high and low groups.

7 Trajectory Analysis

The monocle (version 2.22.0) R package was used to detect the developmental trajectory and branching states of epithelial cells. The "differentialGeneTest" function was used to filter genes which define the trajectory (qvalue < 0.05). Dimensionality reduction was performed using the "DDRTree" method, and cells were ordered by "orderCells" function. The HALLMARK_EPITHELIAL_MESENCHYMAL_ TRANSITION gene set from the MSigDB database was obtained to calculate the epithelial-mesenchymal transition (EMT) enrichment scores of cells, which were then plotted as box plots for each state group. Using the CytoTRACE (version 0.3.3) R package, we predicted the differentiation scores of cells and visualized them on the monocle trajectory. We assumed that dedifferentiation and transition phenotypes occur simultaneously. Therefore, based on the distribution of cell subtypes, we designated the state with lower EMT scores and higher differentiation scores as the root, and then generated a pseudo-temporal trajectory. According to the derived CytoTRACE scores, cells were divided into two groups: the high-scoring group (top 25%) and the low-scoring group. Cells with high scores were considered as primary tumor cells. To identify significant genes regulated along the CRC malignant progression trajectory, the GeneSwitches (version 0.1.0) R package was used. GeneSwitches is a statistical framework based on logistic regression that can be used to screen for the order of appearance of surface proteins, study how cell functions are turned on and off over time, and compare the order of switch genes in branch trajectories. First, single-cell gene expression and monocle results were input into GeneSwitches for analysis. Then, the "binarize_exp" function was used to binarize gene expression levels into on or off states, setting a fixed cutoff value of 0.2. For each gene, the "find_switch_logistic_fastglm" function computed the switching time and associated confidence level. The top gene with the best fit and the top 20 transcription factors and surface protein genes were visualized using the "plot_timeline_ggplot" function. For gene ontology analysis, genes with confidence levels greater than 0.01 were input into the "find_switch_pathway" function, and enrichment analysis was performed using default parameters.

8 Cell–cell interaction network analysis

To evaluate cell-cell interactions, the R package "CellChat" (version 1.6.1) was utilized to infer cell-cell communication networks. The "createCellChat" function was employed on the raw count matrix and cell type annotations to create a CellChat object, followed by filtering to obtain significant interactions. The strength of these interactions was quantified by calculating the average log-normalized counts of ligand genes in the source cell type and receptor genes in the target cell type. Cell-cell interactions and receptor-ligand pairs were visualized using CellPhoneDB (www.cellphonedb.org). This analysis method examines differences in cell-cell communication networks, revealing signaling dynamics among malignant epithelial cells, stromal cells, and immune cells in the tumor microenvironment.

9 Statistical analysis

All statistical analyses were performed using R version 4.1.1 (https://www.r-project.org/). For all statistical tests, a two-sided P value less than 0.05 was regarded statistically significant.
