# Supplementary material for: Single-Cell Transcriptomics Reveals Cellular Heterogeneity and Drivers in Serrated Pathway-Driven Colorectal Cancer Progression
Source: Int J Mol Sci. 2024 Oct 11;25(20):10944. doi: 10.3390/ijms252010944 (PMC11507054; doi:10.3390/ijms252010944)
Supplement: Supplementary file 1 [file ijms-25-10944-s001.zip › Supporting figures.doc.pdf]

Supplementary Figures

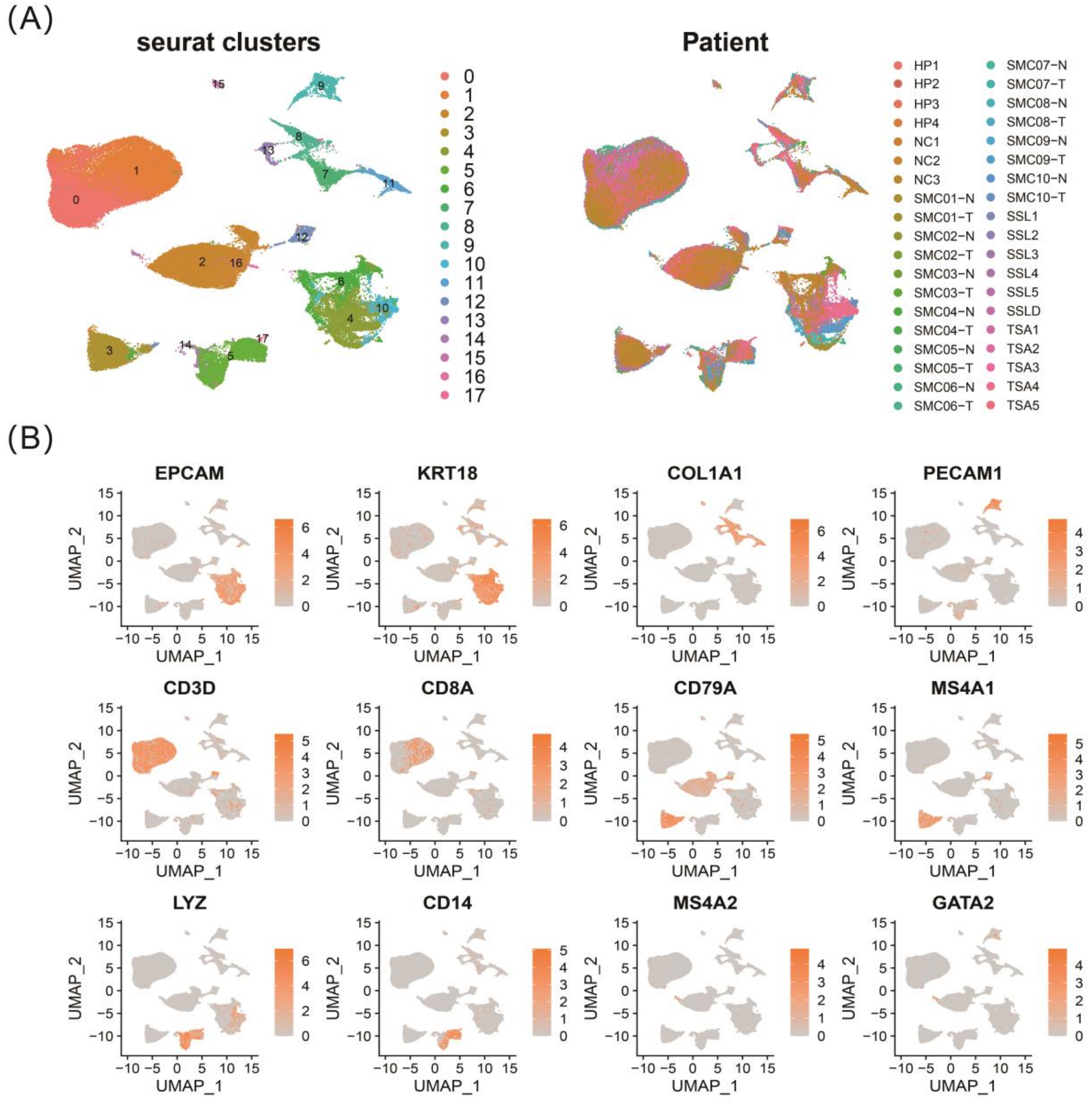

Figure S1. Annotation of Major Cell Types

(A) Clustering of all cells, grouped into 18 clusters (left); mapping of sample origin in the UMAP plot (right). (B) Mapping of marker gene expression levels for cell types in the UMAP plot.

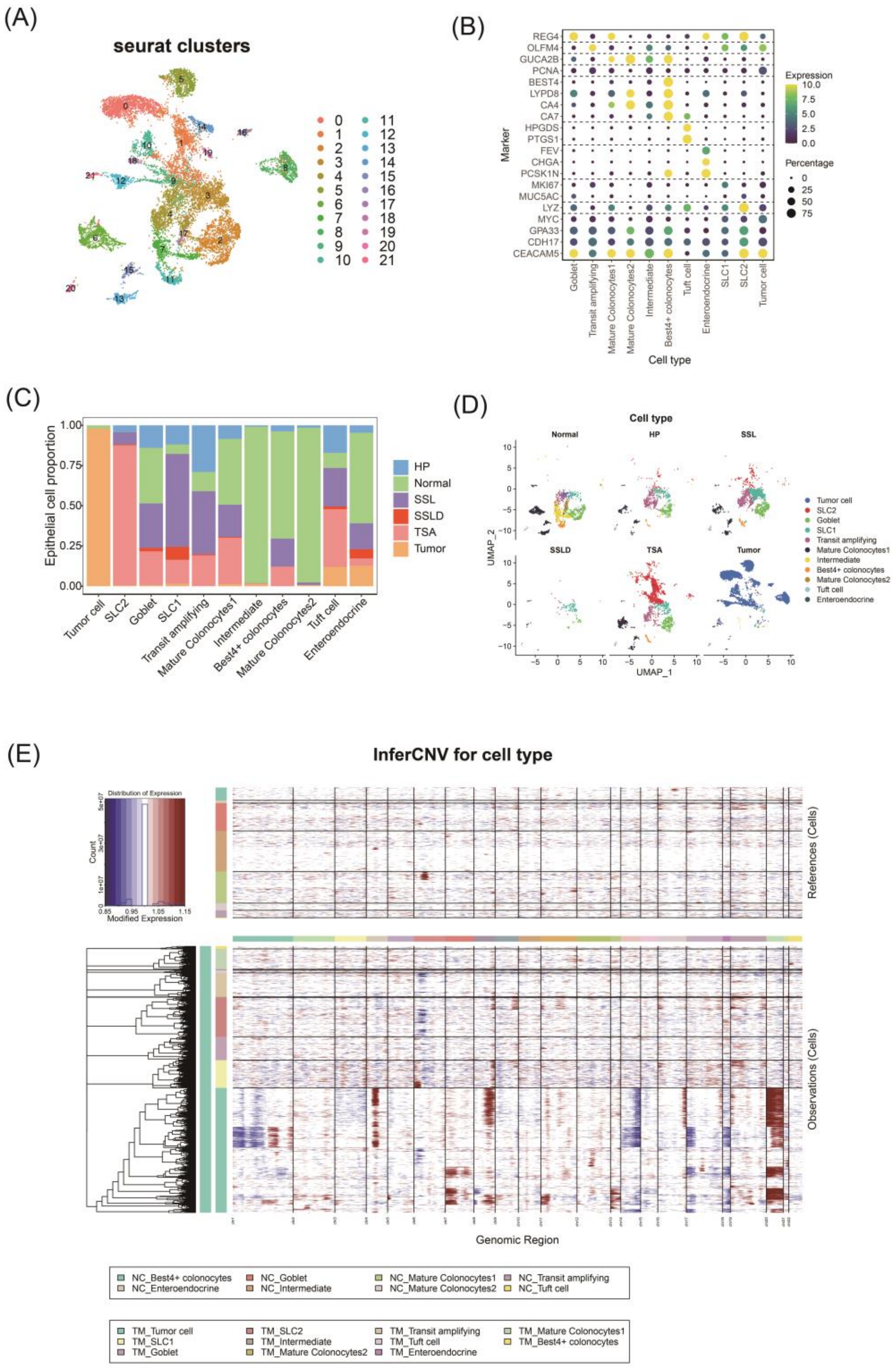

Figure S2. Exploration of epithelial cell subtype heterogeneity

(A) Clustering of epithelial cells, grouped into 22 clusters. (B) Marker genes used for annotating each cell type. (C) Bar chart showing the proportion distribution of tissue origin in epithelial cell subtypes. (D) UMAP dimensional reduction plot of cell subtypes by tissue origin. (E) InferCNV analysis. Hierarchical heatmap showing large-scale CNVs in different epithelial cell subtypes to identify malignant cells.

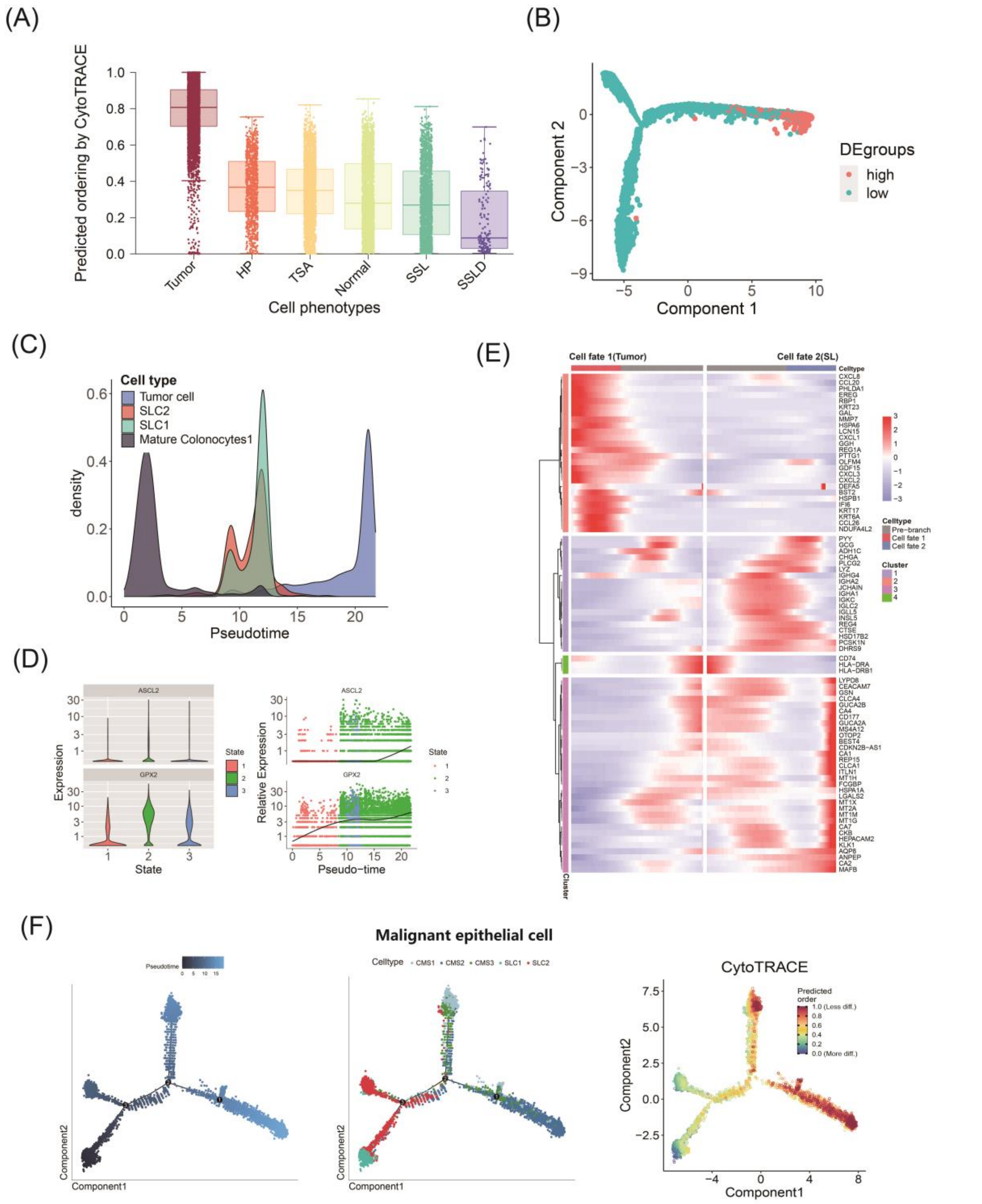

Figure S3. Functional Exploration in Epithelial Cell Trajectory Analysis

(A) CyTRACE scores by tissue type. (B) High plasticity cells (red) with CytoTRACE scores >0.75. (C) Cell density across pseudotime. (D) Gene expression distribution for ASCL2 and GPX2 across 3 states (left); their expression changes throughout pseudotime (right). (E) Heatmap of 80 branch-dependent genes across 3 states identified by BEAM. (F) Monocle trajectory plot of SLC1, SLC2, and Tumor cells (left); celltype distribution along the trajectory (middle); CytoTRACE scores mapping along the trajectory (right).

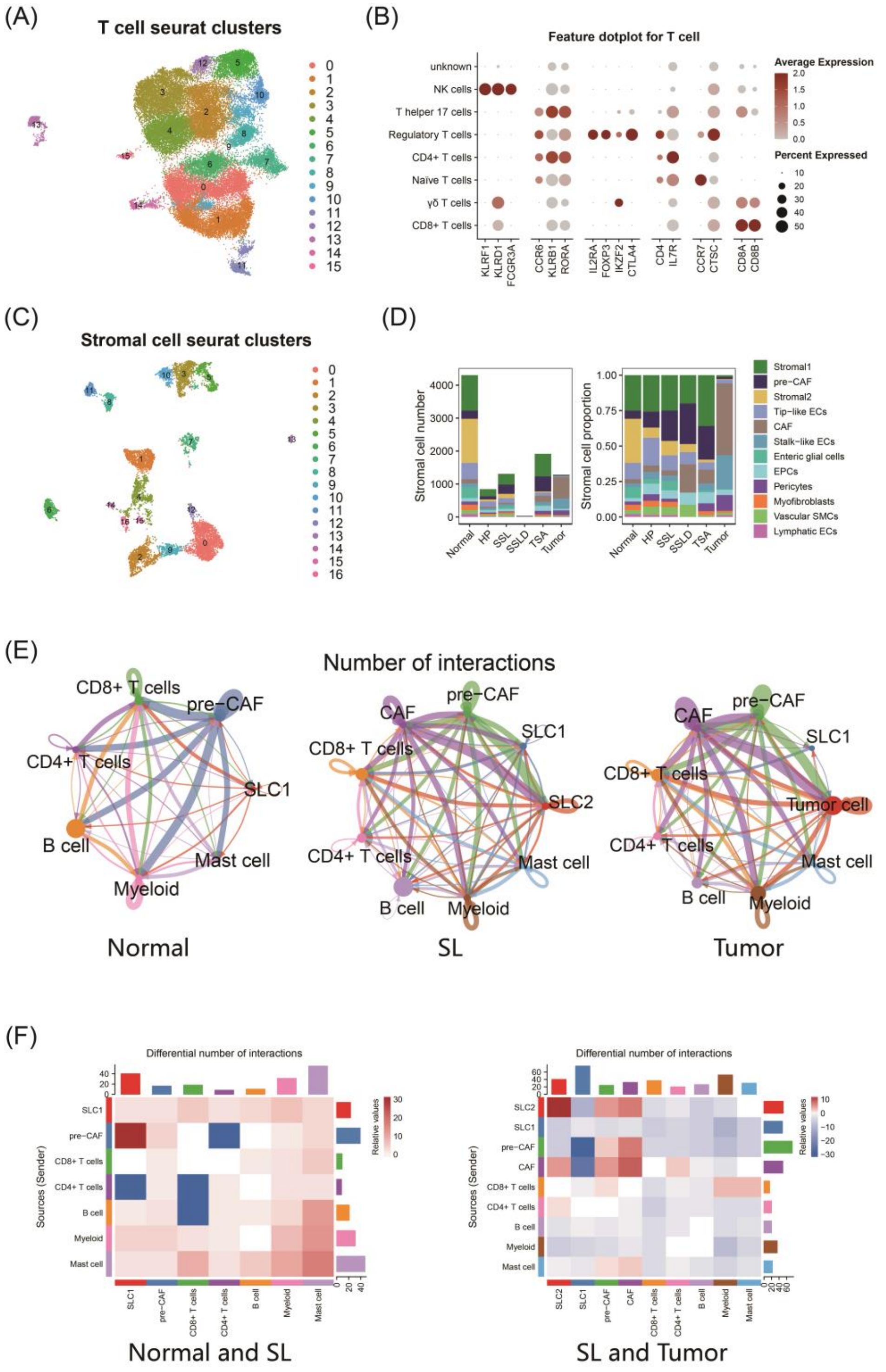

Figure S4. Cellular Atlas and Cell Communication in the Tumor Microenvironment

(A) T cell clustering into 16 groups. (B) Bubble plot of marker gene expression for T cell subtypes. (C) Stromal cell clustering into 17 groups.(D) Bar plot of stromal cell subtype distribution across tissue sources.(E) Intercellular communication counts in Normal, SL, and Tumor samples.(F) Heatmaps comparing intercellular communications: Normal vs. SL (left) and SL vs. Tumor (right); colors indicate communication differences (red: more in SL, blue: fewer in SL).
